# The Theory and Practice of Medicine, Etc.

**Published:** 1894-02

**Authors:** 


THE THEORY AND PRACTICE OF MEDICINE PREPARED FOR STUDENTS AND PRACTITIONERS. By James T. Whittaker, M. D., LL. D., Professor of the Theory and Practice of Medicine in the Medical College of Ohio; Lecturer on Clinical Medicine at the Good Samaritan Hospital; Fellow of the College of Physicians of Philadelphia; Member of the Association of American Physicians, of the American Academy of Medicine, and of the American Medical Association. With a Chromo-Lithographic Plate and three hundred Engravings. Octavo, 840 pages; more than three hundred Engravings and one Chromo-Lithographic Plate. Extra muslin, price, $5.75; leather, price, $6.50. New York: William Wood & Company.

In the preparation of this book the author has bestowed much work on the infections and infectious diseases. He believes that a thorough knowledge of the causes of disease is of the greatest importance in arriving at a knowledge of the best course to pursue in its treatment, and more especially in its prevention.

The book is divided into two parts. Part first is devoted to "General Diseases," under the sub-headings of "Infection" and "Parasites." Part second of the book is devoted to the consideration of "Diseases of Organs," under the special subjects of "Diseases of the Organs of Digestion," "Diseases of the Organs of Respiration," "Diseases of the Organs of Circulation," "Diseases of the Genito-Urinary System," and "Diseases of the Nervous System."

Throughout the entire work, bacteriology is given a prominent place, and the book may be said to be written by an enthusiastic bacteriologist. The prominence that the author gives to the causation and diagnosis of disease, especially in so far as it relates to the infections, constitutes one of the most valuable features of the book, and in this feature it surpasses most, if not all, of our older text-books on the theory and practice of medicine. This book will become especially popular with the medical student and the young practitioner, as it is written in a style so plain and easy of comprehension, and the rules laid down for the management of cases of disease are so positive and pointed that confidence in the mind of the practitioner will be established at once.

The publishers are to be congratulated on securing a book of much excellence, and they are to be commended for the splendid style in which the book is gotten out.

